# Management of Severe Hidradenitis Suppurativa

**DOI:** 10.7759/cureus.13483

**Published:** 2021-02-22

**Authors:** Ju Hee Katzman, Maryam Tahmasbi, Masoumeh Ghayouri, Sowmya Nanjappa, Michael C Li, John Greene

**Affiliations:** 1 Infectious Disease, Moffitt Cancer Center, Tampa, USA; 2 Pathology and Cell Biology, University of South Florida Morsani College of Medicine, Tampa, USA; 3 Pathology, Moffitt Cancer Center, Tampa, USA; 4 Infectious Disease, University of South Florida Morsani College of Medicine, Tampa, USA; 5 Internal Medicine, Moffitt Cancer Center, Tampa, USA

**Keywords:** hidradenitis suppurativa, crohn’s disease, anal fistula, verneuil’s disease

## Abstract

Hidradenitis suppurativa (HS) is a devastating and disfiguring disease of the skin involving the terminal follicular epithelium within the apocrine-gland-bearing skin. We present an interesting case of a 58-year-old female who presented with a 10-year history of refractory HS of the gluteal, perineal, perianal, and groin region. She had been chronically treated with multiple antibiotics in the past with no improvement. The patient subsequently underwent surgical local excision with complex closure. Medical management alone may not be optimal, especially in refractory disease. Early and aggressive surgical intervention and interdisciplinary approach are needed for patients with chronic and advanced stage of HS.

## Introduction

Hidradenitis suppurativa (HS) derived from the Greek word hideos meaning sweat and aden meaning gland is a chronic, recurrent, suppurative, cutaneous disease, manifested by abscesses, fistulating sinus tracts, and scarring [1]. Synonyms of HS include apocrinitis, acne inversa, and pyoderma fistulans significa. There has been significant debate around the pathological features of HS. This debate focuses on whether the primary event relates to an inflammatory process of the apocrine duct or whether follicular occlusion is integral to the initiating process. However, it is now believed that HS conforms to a disorder of terminal follicular epithelium within apocrine-gland-bearing skin, but apocrine involvement does not appear to be a primary event in the majority of cases. Rather, follicular occlusion is thought to be the inciting event in disease development [1].

The disease is expressed by a variety of clinical features. It affects apocrine-gland-bearing skin; the axillae are most often involved, followed by the anogenital region. Uncommon sites include the areola of the breast, the submammary fold, the periumbilical skin, the scalp, and the zygomatic and malar areas of the face, the buttocks, the thighs, and the popliteal fossa [2]. We present a unique case of refractory HS involving extensively the perianal, perineal, groin, and gluteal areas. This case was previously presented as a meeting abstract at the 2017 Hospital Medicine Meeting on May 1-4, 2017.

## Case presentation

**Figure 1:**
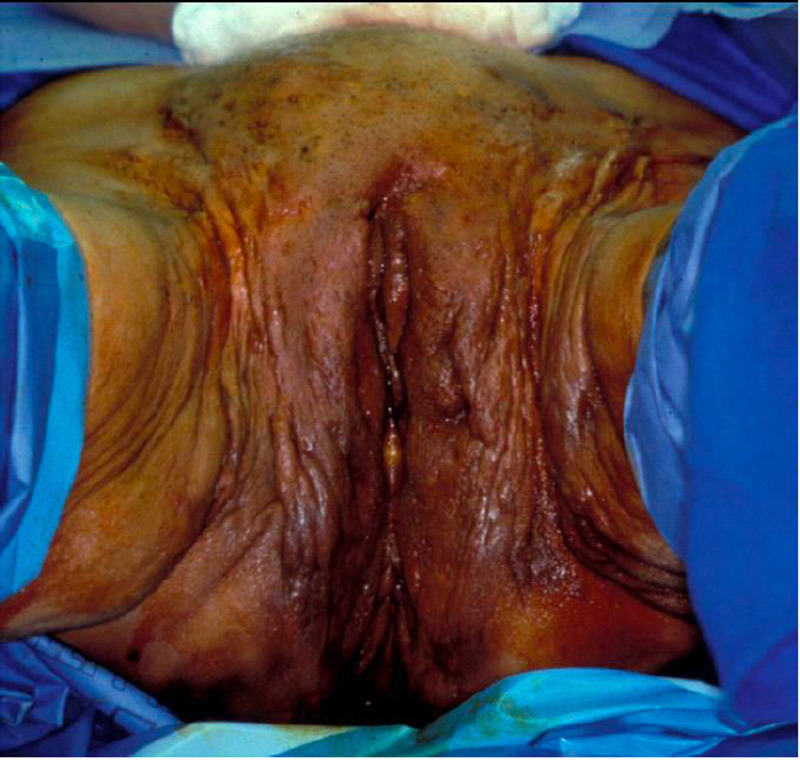
Preoperative image of the external genitalia of a comparable case. Marked scarring and fistulous tracts were evident in the pubis, vulva, and groin.

**Figure 2:**
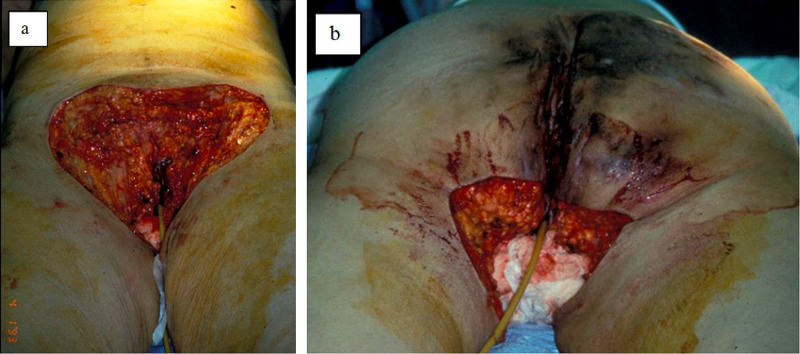
Postoperative image of a comparable case. Patient underwent modified radical vulvectomy, excision of left perianal HS, and advancement skin flap closure. HS, hidradenitis suppurativa

**Figure 3:**
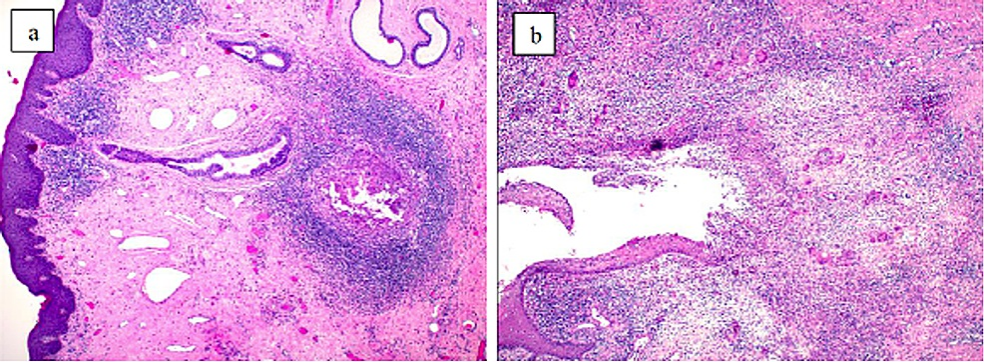
Histopathology of HS (hematoxylin-eosin, original magnification ×4). (a) Hair follicle with desquamated keratin, surrounded by dense inflammatory infiltrates; (b) suppuration may extend into the adjacent connective tissue, where there may be a foreign body-type reaction of histiocytes and giant cells due to the keratin. HS, hidradenitis suppurativa

**Figure 4:**
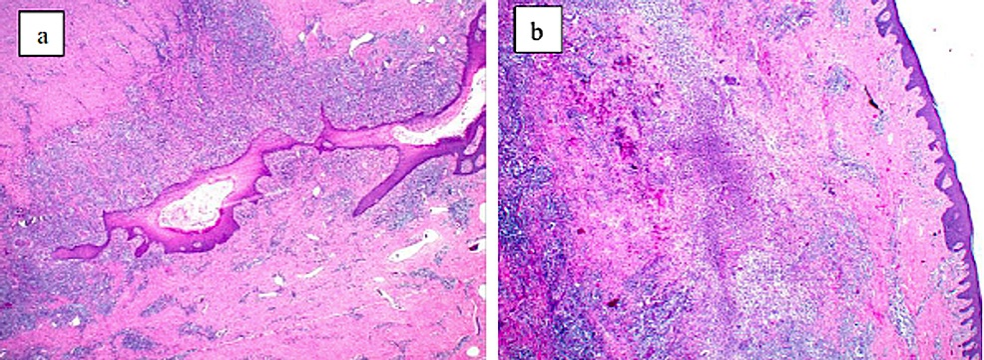
Histopathology of HS (hematoxylin-eosin, original magnifications ×2). (a) Sinus tract with heavy neutrophilic or mixed inflammatory infiltrate; (b) marked suppuration and frank abscess formation.

A 58-year-old Caucasian female presented with a 10-year history of painful HS of the gluteal, perianal, perineal, and vulvar areas with increasing drainage and scar formation. She denied having fevers or chills. Treatment with amoxicillin, doxycycline, sulfamethoxazole-trimethoprim, and fluconazole was unsuccessful. Her past medical history included Crohn’s disease, hemorrhoids, pilonidal cysts, irritable bowel syndrome, coronary artery disease, and hyperlipidemia. She also had rectal fistulas repaired four times. Colonoscopy showed reactive lymphoid infiltrate with melanosis coli. No treatment was given for Crohn’s disease. No stool incontinence or feces through the vagina was noted. She continued to smoke cigarettes daily. There was no family history of autoimmune diseases. Upon physical examination, marked scar tissue with some purulent drainage was noted at the perineal, perianal, and inguinal area (Figures 1, 2 show a clinical image of a comparable case). Her body mass index was 22.1 kg/m^2^. Her surgery included wide local excision with complex closure involving 20 cm of both buttocks as well as 15 × 8 cm of the right vulva including the mons pubis and 12 × 4 cm of the left vulva (Figure 2). The surgery was performed by plastic, gynecology, and gastroenterology surgery departments. Postoperatively, she developed erythema over the right medial thigh into the right groin which did not improve on doxycycline but eventually responded to the addition of vancomycin. Methicillin-resistant *Staphylococcus aureus* (MRSA) nasal screen was negative. She remained afebrile. Her white blood cell count was as high as 12.7 cells/cc but normalized after surgery. Histopathology of the excisional areas demonstrated chronic granulomatous inflammation with sinus formation consistent with HS and negative for malignancy (Figures 3, 4). She was discharged with a one-week course of intravenous vancomycin and then a one-week course of oral trimethoprim-sulfamethoxazole. At one-year follow-up, she complained of worsening chronic pain at the perianal area. She had developed few small pustules on the right perianal area which was tender to palpation. She was given a one-week course of oral amoxicillin clavulanate and the recurrence resolved.

## Discussion

Several clinical features of our patient were consistent with the current incidence and clinical manifestations of HS. The disease incidence usually peaks during the third decade of life and is more common in females at a ratio of 3:1 according to a recent population-based study [3]. Comorbidities that are significantly associated with HS include gender, obesity, metabolic syndrome, and pilonidal disease [3-6]. Smoking has been associated with worse disease and cessation has been shown to be integral in disease control [5]. Involvement of the groin, perineal, and perianal areas show mixed characteristics of the disease seen among both genders. Perianal and perineal involvement is more commonly seen among men while axillary, upper anterior torso, and groin involvement is more common among women [4]. The differential diagnoses in this setting include follicular pyodermas, acne vulgaris, intergluteal pilonidal disease, skin manifestation of Crohn’s disease, and granuloma inguinale. HS presenting among adolescents has been reported as PAPASH syndrome (pyogenic arthritis, pyoderma gangrenosum, acne, and suppurative hidradenitis), a condition associated with the *PSTPIP1 *gene mutation. Another closely related PASH syndrome (pyoderma gangrenosum, acne, and suppurative hidradenitis) does not involve this gene mutation [7]. Patients with inflammatory bowel disease have been shown to be more predisposed to develop HS than the general population [8]. Multiple case reports have demonstrated this coexistence, and up to 17% of patients with Crohn’s disease and up to 8% of patients with ulcerative colitis have reported symptoms suggestive of HS [8]. The clinical and histologic similarities between the two diseases suggest a relationship; however, further investigation is needed [8]. The presence of T-lymphocytes expressing the same biomarkers such as CD4+, CD161+, CD4+, and IL-17 may play a pathogenic role in patients with inflammatory bowel disease and concurrent HS [9]. The microorganisms involved in the pathogenesis of chronic HS include anaerobic bacteria (gram-positive cocci and gram-negative rods such as *Prevotella*, *Porphyromonas, *and *Fusobacterium *spp.), milleri group streptococci (*S. angionsus* and *S. constellatus*), and anaerobic actinomycetes (*A. turicensis*, *A. radingae*, *A. neuii*, and *Actinobacculum schaalii*) [10]. A recent review showed that HS lesions are uncommonly sterile and major skin pathogens such as *S. aureus* and *S. pyogenes* do not play an important role. The commensal *S. lugdunensis* have been implicated in the early manifestations of HS such as nodules and abscesses [10]. In essence, the important microorganisms for HS originate from the gut and oral microbiota. Actinomycetes, for example, have been associated with difficult to treat or relapsing skin abscesses. These pathogenic organisms are capable of counteracting the host immune system, promoting inflammation, and extending lesions in deep-seated HS. This may also reflect the relative ineffective biotherapies in severe HS [11].

Early lesions of hidradenitis show hyperkeratosis of the terminal hair follicle, with subsequent occlusion of the follicle and dilatation with stasis in apocrine and exocrine glands. As the disease progresses, extensive perifolliculitis and spongiform infundibulofolliculitis are seen, combined with cystic epithelium-lined structures containing hair shafts. The final stage is characterized by a dermis with an inflammatory cell infiltrate, granulation tissue, giant cells, subcutaneous abscesses, and sinus tracts. The inflammatory process involves a localized cell-mediated reaction composed of lymphocytes, neutrophils, and histiocytes, with T-cells comprising a majority of the lymphocytic response. Cystic poral occlusions are commonplace along with fibrosis and, less frequently, primary apocrine involvement. Given the frequency of these occlusions, hidradenitis may be better classified as a follicular disease. In vulvar hidradenitis, apocrine glands are typically distantly located from infected areas, with eccrine glands being predominant in the affected regions [12]. Thus, inflammation and destruction of apocrine glands seem to be the sequelae of the inflammatory process [12]. Key pathologic features include presence of apocrine glands, suppurative folliculitis with abscess formation, sinus tracts with suppurative and granulomatous inflammation, granulation tissue, and inflammation “spilling over” to involve apocrine glands [13].

The simplest, most widely used staging system for HS is known as the Hurley’s criteria (Table 1) [14]. It is used to guide treatment principles based on the severity of the disease, although it is not useful for measuring clinical trial interventions [6]. According to this criteria, only 1% of patients progress to stage III. In addition to Hurley’s criteria, there are several other staging systems developed such as the more detailed but time-consuming Modified Sartorius Score, the HS-Physician’s Global Assessment, and the HS-Severity Index [6].

**Table 1:** Hurley’s Criteria for classification of HS [14]. HS, hidradenitis suppurativa

Stages	Clinical features
I	Single or multiple abscesses, without sinus tracts or cicatrization
II	Single or multiple recurrent abscesses, with tract formation and cicatrization and widely separated lesions
II	Multiple interconnected abscesses and tracts across the entire area, with diffuse or near-diffuse involvement

Medical management combined with a surgical approach provides the best chance of cure for patients with refractory HS, with surgery providing significant patient-reported improvement in quality of life [15]. First-line medical therapy for newly diagnosed patients typically includes the use of clindamycin and rifampicin, a combination which modulates T-lymphocytes and can interfere with HS biofilm formation [14]. Likely due to the many androgen receptors in apocrine sweat glands, hormonal therapies have also demonstrated clinical use, such as finasteride, a 5-alpha reductase inhibitor, and spironolactone, which possess anti-androgenic activity [16]. Although these hormonal therapies are not recommended for males, the female predominance of HS may prove beneficial when treating HS. In addition to antibiotic and hormonal therapies, the medical management for severe HS with proven efficacy is infliximab and alternatives include adalimumab, etanercept, and acitretin, with the first three being anti-tumor necrosis factor alpha inhibitors and the fourth a vitamin A derivative [6,14,17]. Other systemic biologics that have been tried successfully include the use of IL-23/23 inhibitors, such as ustekinumab [18]. The elimination of the nidus of infection, sinus tracts, and scars leads to prevention of disease recurrence and a chance of cure. Recurrence rates are reduced with radical excision compared to incision and drainage. Incision and drainage relieves acute pain due to abscess; however, due to the high recurrence rate, this practice should be limited [17]. In addition to medical and surgical management, prevention and controlling risk factors are also important such as smoking cessation, weight loss, and skin hygiene. Despite the debate regarding the pathophysiology as well as medical and surgical management of HS, it is widely acceptable to opt for early surgical intervention among patients with refractory and severe HS given its physical and psychosocial morbidities [17]. HS is associated with shame, isolation due to fear of stigmatization, high degree of morbidity due to pain, higher risk of suicide, and poor quality of life correlated with number of HS lesions, severity, and duration [19]. Notably, involvement of the anogenital region affects the quality of life the most [17]. HS is also associated with the development of squamous cell carcinoma and represents a poor prognosis [20].

## Conclusions

HS is a chronic, suppurative, and scarring condition of the areas of skin which bear the apocrine glands. The microorganisms involved in the pathogenesis likely involve the oral and gut flora than normal skin flora. Patients presenting with signs of advanced stage of HS need early aggressive surgical intervention as medical management, largely centered around immunomodulation, is not optimal in controlling the complications.
